# Dosimetric Performance of Poly(vinyl alcohol)/Silver Nanoparticles Hybrid Nanomaterials for Colorimetric Sensing of Gamma Radiation

**DOI:** 10.3390/nano12071088

**Published:** 2022-03-26

**Authors:** Phasit Petisiwaveth, Rujira Wanotayan, Nuanpen Damrongkijudom, Sumalee Ninlaphruk, Sumana Kladsomboon

**Affiliations:** 1 Department of Radiological Technology, Faculty of Medical Technology, Mahidol University, Nakhon Pathom 73170, Thailand; phasit.peti@gmail.com (P.P.); rujira.wan@mahidol.edu (R.W.); nuanpen.dam@mahidol.ac.th (N.D.); 2 High-Dosimetry Calibration Laboratory, Office of Atoms for Peace, Bangkok 10220, Thailand; sumalee.n@oap.go.th

**Keywords:** radiation sensors, dosimetry, poly(vinyl alcohol), silver nanoparticles, gamma radiation

## Abstract

A colorimetric liquid sensor based on a poly(vinyl alcohol)/silver nanoparticle (PVA/AgNPs) hybrid nanomaterial was developed for gamma radiation in the range of 0–100 Gy. In this study, gamma rays (Cobalt-60 source) triggered the aggregation of AgNPs in a PVA/silver nitrate (AgNO_3_) hybrid solution. The color of this solution visibly changed from colorless to dark yellow. Absorption spectra of the PVA/AgNPs solution were analyzed by UV-Vis spectrophotometry in the range of 350–800 nm. Important parameters, such as pH and AgNO_3_ concentration were optimized. The accuracy, sensitivity, stability, and uncertainty of the sensor were investigated and compared to the reference standard dosimeter. Based on the spectrophotometric results, an excellent positive linear correlation (*r* = 0.998) between the absorption intensity and received dose was found. For the accuracy, the intra-class correlation coefficient (ICC) between the PVA/AgNPs sensor and the standard Fricke dosimeter was 0.998 (95%CI). The sensitivity of this sensor was 2.06 times higher than the standard dosimeter. The limit of detection of the liquid dosimeter was 13.4 Gy. Moreover, the overall uncertainty of this sensor was estimated at 4.962%, in the acceptable range for routine standard dosimeters (<6%). Based on its dosimetric performance, this new PVA/AgNPs sensor has potential for application as an alternative gamma sensor for routine dose monitoring in the range of 13.4–100 Gy.

## 1. Introduction

Gamma radiation is a high energy ionizing radiation in the form of electromagnetic waves with the ability to penetrate materials. Gamma rays are routinely used in diverse applications, including polymer synthesis [1], food irradiation [2], and medical radiation [3]. The dose range used in each application can vary from a few mGy up to several kGy. Adverse effects of ionizing radiation may result from under-exposure when used for food safety [4] and over-exposure when used for human health [5]. Therefore, it is extremely important to accurately control the received radiation dose on a material. Common radiation sensors (or dosimeters) used for the determination of radiation dose include ionization chambers, semiconductors, chemical-based sensors, thermoluminescence, etc. However, most of the traditional methods do not provide the ideal characteristics of a dosimeter, which is a minimum requirement of manipulation, reasonable cost, and high radiation sensitivity [6]. Thus, development of a highly sensitive, cost-effective, convenient, and nontoxic gamma radiation sensor remains a goal, despite several decades of research.

Recently, nanoscience and nanotechnology have shown that the development of a novel radiation dosimeter at nano scale is possible, in particular a colorimetric-based dosimeter. A change of nanoparticle color after irradiation allows the visual estimation of radiation dose [7,8]. Several metallic nanoparticles have been used for the development of color change, for example, silver (Ag) [9], gold (Au) [10], copper (Cu) [11], bismuth (Bi) [12], and nickel (Ni) [13]. Ag nanoparticles (AgNPs) have often been the material selected due to their biocompatibility [14] and unique physiochemical properties [15]. AgNPs are used in several diagnostic [16], therapeutic [17], and industrial applications [18,19].

Among colorimetric dosimeters, metal nanoparticles stabilized by polymer matrix have been widely studied due to their advanced optical, catalytic, electronic, and electro-chemical properties [20,21,22]. The synthesis of nanoparticles in a polymer matrix by an irradiation process can induce color change in the system through surface plasmon resonance [23]. Several polymer matrices, namely gelatin, poly(vinyl alcohol) (PVA), poly(vinylpyrrolidone), poly(alanine), and poly(acrylamide) were studied with a focus on factors such as radiation dose, pH, alcohol, AgNO_3_ concentration, and stabilizer concentration [23,24,25,26,27]. Soliman et al. [24] introduced a silver-gelatin based radiochromic gel dosimeter system. The dosimeter showed a linear response (*r*^2^ = 0.9995) upon Cs-137 irradiation up to 100 Gy. An enhancement of radiation sensitivity by increasing the AgNO_3_ concentration was observed up to 250 mM in this study. Other related parameters such as pH value, stabilizer concentration, and isopropanol content were studied by Tadros et al. [23]. Their study showed that increases of precursor concentration enhances the radiation sensitivity of Ag nanoparticle gel dosimeters up to an optimal point. Moreover, the sensitivity of this system was enhanced with increases of precursor concentration. The highest sensitivity (7.44 × 10^−^^3^ Gy^−^^1^) was found at optimized conditions (100 mM of AgNO_3_ concentration, 4% gelatin content, and 20% of iso-propanol) in this system. The reliability of dose measurement in the studies was reported as overall uncertainties of 4.56% and 5.16%, respectively [23,24].

The matrix based on PVA is recommended and widely used for biomedical application due to its high effectiveness, stability, and safety [15] for both low and high doses of radiation. In addition, the PVA matrix provides a high degree of flexibility [25] and water solubility [26], as well as being environmentally friendly [27]. Merkis et al. [28] introduced a AgPVA film-based dosimeter for low dose application. The AgPVA film showed good sensitivity to x-ray in the range of 0 to 1 Gy. The highest radiation sensitivity of the AgPVA film-based method was reported as 0.34 Gy^−^^1^ up to 1 Gy, then decreasing to 0.15 Gy^−^^1^ as the radiation dose increased. Based on their study [29], increases of AgNO_3_ concentration (0.21 to 1.01 wt%) enhance the radiation sensitivity of the film from 0.20 to 0.34 Gy^−^^1^ in the range of up to 1 Gy. Moreover, the effect of additives such as glycerol, ethanol and isopropanol were demonstrated in the study. These results show the dependence of the radiation sensitivity of the PVA/Ag system on the medium of its environment. Therefore, the related parameters that influence the properties of a PVA/Ag-based dosimeter must be optimized in order to maximize sensitivity and accuracy. 

In this study, PVA/AgNPs hybrid nanomaterials were introduced as an alternative liquid radiation sensor for Gy level application (0–100 Gy). Key parameters such as pH and AgNO_3_ concentration were optimized. The optical properties of PVA/AgNPs solutions were characterized with the naked eye and a UV-vis spectrophotometer. Dosimetric performance (accuracy, sensitivity, stability, and uncertainty) was determined and compared to the standard Fricke dosimeter.

## 2. Materials and Methods

### 2.1. Chemicals

All chemicals were analytical reagent (AR) grade. PVA (Mw ≈ 93,500) (Sigma Aldrich, Burlington, MA, USA), ultrahigh purity AgNO_3_ (Mw = 169.87 g/mol; Merck, Kenilworth, NJ, USA), and ultrapure water 18.2 MΩ.cm (Millipore, Burlington, MA, USA) were used for the preparation of the PVA/AgNPs solutions. The pH values were adjusted using hydrochloric acid (HCl) or sodium hydroxide (NaOH) (both Merck, Darmstadt, Germany). To avoid early AgNO_3_ photoreduction, all procedures were performed under dark conditions [24]. The standard Fricke dosimeter was provided by the High-Dose Dosimetry Calibration Laboratory (HDCL), Office of Atoms for Peace, Bangkok, Thailand.

### 2.2. Preparation of PVA/AgNPs Liquid Dosimeter 

A stock solution of 5% (*w/w*) PVA was prepared by dissolving 5 g of PVA powder in 95 mL of distilled water. To make a homogenous polymer solution, the PVA solution was gently stirred using a hot plate magnetic stirrer under controlled temperature (approximately 80–90 °C) for 7 h. Then the solution was allowed to cool at room temperature and kept in the dark. The PVA/AgNPs solution was prepared by mixing 10 mL of fresh AgNO_3_ solution in 90 mL of PVA solution and stirring at room temperature for 30 min. The effect of the pH was studied. The pH of PVA/AgNPs with 10 mM AgNO_3_ was adjusted to 3, 4, 5, 5.5, and 6 by adding a few drops of 1 M of HCl or 1 M of NaOH solution. The effect of AgNO_3_ concentration was studied by adjusting the total AgNO_3_ concentration to 1, 5, 10, 50, and 100 mM at pH 5. These solutions were prepared by adding AgNO_3_ concentrations into the PVA solution in a dropwise manner. The pH values were confirmed using pH test strips.

### 2.3. Irradiation Technique

All PVA/AgNPs solutions were exposed to gamma rays within semi-micro plastic cuvettes at room temperature using a Co-60 source (Gamma Cell 220). The irradiation was performed in the range of 0–100 Gy at a dose rate of 0.532 Gy s^−1^. The irradiator was calibrated using a standard Fricke dosimeter (ASTM 51026, 2015). The solutions were placed in poly(methylmethacrylate) holders at the middle position of the cell during irradiation in order to ensure the homogeneity of radiation, adequate depth dose, and an electronic equilibrium condition. 

### 2.4. Characterization Techniques

UV-Vis spectrophotometry was applied to analyze the strong oscillations of the AgNPs interacting with the electromagnetic field at specific wavelengths. The absorbance spectra and characteristic peaks (λ_max_) of the PVA/AgNPs solutions were determined using a Lambda 650 UV-Vis spectrophotometer. Absorption spectra operated in the range of 350 to 800 nm. In order to normalize the results, all solution samples were transferred to standard cuvettes before measurement. To investigate dose response, the absorbance spectra of irradiated solutions were measured after 1 h of incubation. The dose response was expressed in terms of change in λ_max_ (430 nm) as a function of dose. 

### 2.5. Statistical Analysis

For each measurement, the absorption spectra of PVA/AgNPs solutions were determined in triplicate. The relationship between the change of λ_max_ and the radiation dose of the solution was assessed by linear regression, using Pearson’s correlation coefficient (r) and slope. Accuracy was evaluated by comparing the dose measured by the PVA/AgNPs method to that of the standard reference method (Fricke dosimetry). The degree of agreement between the two methods was determined by intraclass correlation coefficient (ICC) based on mean-rating (*n* = 3), absolute-agreement, and a 2-way mixed-effects model [30,31]. 95% confident intervals were determined using the SPSS statistical package version 18 (SPSS, Chicago, IL, USA).

## 3. Results and Discussion

### 3.1. Principle of PVA/AgNPs for Radiation Sensor

**Figure 1:**
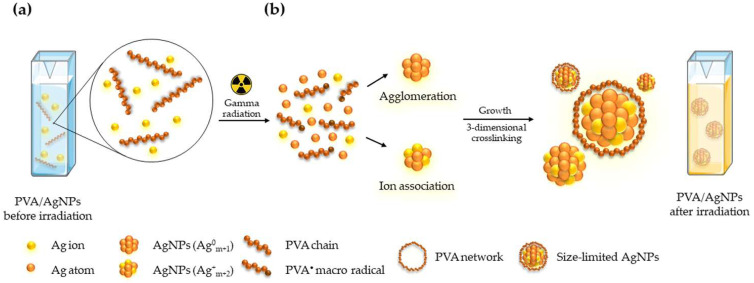
Principle of the colorimetric sensor based on a hybrid PVA/AgNPs: (**a**) before and (**b**) after gamma irradiation.

**Figure 2:**
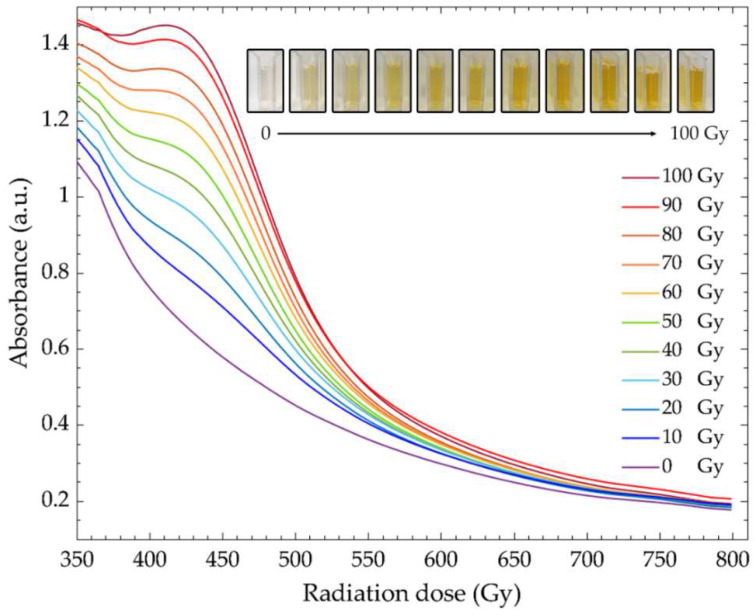
Absorption spectra and digital photographs of PVA/AgNPs solution before and after irradiation with gamma rays up to 100 Gy.

The proposed mechanism of a PVA/AgNPs sensor for colorimetric radiation sensing is illustrated in Figure 1. In this system, the presence of PVA played an important role as the stabilizer for AgNPs synthesis [32]. Before irradiation (Figure 1a), silver ions (Ag^+^) and PVA chains remained in a dispersed state with a clear PVA-AgNO_3_ solution. Based on previous studies, absorption spectra of pure PVA [29] and AgNO_3_ [33] solution are absent in the visible region, implying that AgNPs are not formed in AgNO_3_ solutions without PVA. However, the characteristic peak of AgNPs at 430 nm was observed for the irradiated PVA/AgNO_3_ solution (Figure 2). Thus, the presence of AgNPs in this solution was confirmed.

Through irradiation (Figure 1b), the AgNPs and PVA were “tricked” into forming a network. The formation of AgNPs can be explained as two steps: (1) the formation of an Ag nanocluster via agglomeration and/or ion association of Ag atoms, and (2) the restriction of nanoparticle size via polymerization of PVA [34,35,36]. In the first step, the presence of the strong reducing species, i.e., hydrated electrons ($e_{\mathrm{aq}}^{-}$), hydroxyl radicals (${\mathrm{OH}}^{•}$), and hydrogen radicals ($H^{•}$), caused by the radiolysis of water via a photoelectric effect and Compton scattering could reduce metal ions to form zero-valent metal atoms. In this case, the Ag^+^ from AgNO_3_ in an aqueous solution was reduced to a silver atom (Ag^0^). The zero-valent Ag atoms were further agglomerated into AgNPs (Ag^0^_m__+__1_) and then formed into larger AgNPs (Ag^+^_m+2_) by reacting with unreduced Ag^+^ in the solution. The process led to the formation of larger AgNPs in the solution. Next, the increased nanoparticle size was limited by the PVA network in the solution, which acted as a stabilizer of the AgNPs. Formation of the PVA network may be explained in two steps: (1) a PVA macroradical (${\mathrm{PVA}}^{•}$) was formed by a reaction between a PVA chain and$\;{\mathrm{OH}}^{•}\;$in an aqueous solution, and (2) the ${\mathrm{PVA}}^{•}$ interacted with each other to initiate either inter- or intra-molecular crosslinking, forming a three-dimensional network surrounding the AgNPs [36]. This network limited the size of AgNPs by preventing continued agglomeration of AgNPs [31,37]. Consequently, the color of the PVA/AgNPs sensor changed from colorless to yellow after irradiation.

To verify the mechanism of the PVA/AgNPs sensor for gamma ray sensing, the PVA/AgNPs solution (50 mM of AgNO_3_ concentration in 5% (*w/w*) PVA solution) was investigated using digital photographs and UV-Vis spectroscopy (Figure 2). The presence of AgNPs in this system was confirmed by the color change of the sensing solution, that changed from colorless to yellow. The characteristic peak (λ_max_) of AgNPs was observed at 430 nm. As the radiation dose increased, color of the PVA/AgNPs solution changed from colorless to light yellow and to dark yellow. These remarkable changes of solution color confirmed the formation of AgNPs induced by the radiation process [34]. This gradual color change was observed with the naked eye with a minimum threshold of 10 Gy. In addition, the significant increase of λ_max_ was observed at ~430 nm and was associated with the surface plasmon resonance of the AgNPs [38,39]. A slight shift of λ_max_ (from 437 nm to 430 nm) was observed at high doses. This blue shift could be explained by a decrease of AgNPs’ size due to an increase of PVA macroradicals at a high radiation dose inhibiting the growth of AgNPs in the system [40,41]. Moreover, the full width at half maximum (FWHM) of the absorption spectra gradually decreased at high doses, meaning that the homogeneity of particle size increased as the radiation dose increased [42]. Thus, change of color and increased absorption intensity under the influence of radiation were used as indicators for dose assessment of the PVA/AgNPs sensor.

### 3.2. Optimization of PVA/AgNPs Sensor Conditions

Based on previous studies, pH [37,43,44,45,46,47,48] and AgNO_3_ concentration [49,50,51,52] affect the quantity, size, and shape of synthesized AgNPs. Therefore, to standardize the colorimetric and photometric properties of the sensor, pH and AgNO_3_ concentration were optimized.

#### 3.2.1. Effect of pH 

**Figure 3:**
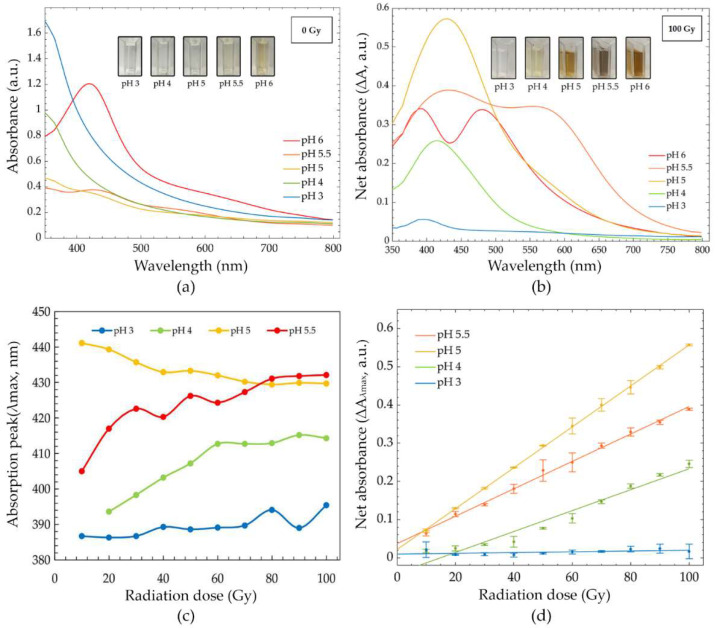
(**a**) Absorption spectra of PVA/AgNPs solution before irradiation (pH 3, 4, 5, 5.5 and 6), (**b**) net absorption spectra after 100 Gy radiation (pH 3, 4, 5, 5.5 and 6), (**c**) absorption peaks (λ_max_) across a range of 0–100 Gy (pH 3, 4, 5 and 6), and (**d**) dose response curves in the range of 0–100 Gy (pH 3, 4, 5, and 5.5).

The effect of pH was studied by varying pH to 3, 4, 5, 5.5, and 6 in PVA/AgNPs solution (10 mM AgNO_3_ in 5% (*w/w*) PVA solution). After sample preparation, the color of the PVA/AgNPs sensors appeared colorless at pH 3 and 4, while they turned a light yellow at pH 5 and 5.5, and yellow at pH6 (Figure 3a). The development of color was associated with changes in the absorption spectra. The characteristic peaks at pH 3, 4, and 5 were not in the visible region, while the characteristic peaks of pH 5.5 and 6 were found at ~426 and ~420 nm (Figure 3a), respectively. These two peaks confirmed the early formation of AgNPs under the influence of pH. This phenomenon could be explained by the reactivity of a reducing agent ($e_{\mathrm{aq}}^{-}$) in the system to high concentrations of protons ($H^{+}$) [44] and indicated the nucleation and growth of nanoparticles [47,48]. The highest absorption intensity was at pH 6, referring to the highest amount of AgNPs synthesis. However, the solution at pH 6 was excluded from further experiments due to the early formation of AgNPs by the effect of alkaline medium, which in turn decreased the ability to estimate radiation dose from its colorimetric properties. Moreover, the availability of high absorption intensity after irradiation might reduce the signal-to-noise ratio of the system, leading to a reduction in accuracy of dose estimation. Figure 3b shows the net absorption spectra of PVA/AgNPs solution after 100 Gy irradiation. These spectra (ΔA = A_i_ − A_0_) illustrate the change of absorption intensity under the influence of the radiation dose. Net absorption can be calculated by the subtraction of the absorption intensity at a dose point (A_i_) from its background absorption (before irradiation; A_0_). Absorption peaks progressively increased for pH 4, 5, 5.5, and 6, while it was unchanged for pH 3. Slight shifts of λ_max_ under the influence of radiation in different pH conditions were observed (Figure 3c). The shifting of the λ_max_ to longer wavelengths (red shift) was observed for pH 3, 4, and 5.5, while the λ_max_ of pH 5 was shifted to a shorter wavelength (blue shift).

Figure 3d shows the dose response curve at different pH values. Dose response curves were plotted as a function of absorption intensity change at the absorption peak (at λ_max_) [∆A_λmax_ = (A_i_ − A_0_)_λmax_] of the receiving dose. Solution peaks were 389, 407, 429, and 432 nm for pH 3, 4, 5 and 5.5, respectively. The linear relationship between absorption intensity and the absorbed dose (0–100 Gy) was plotted. Pearson’s correlation coefficient of simple linear regression for pH 3, 4, 5, and 5.5 were 0.503, 0.976, 1, and 0.998, respectively. High correlation coefficients (>0.9) were found at pH 4, 5, and 5.5. Radiation sensitivities were estimated using the slope of the linear regression models. The sensitivities at pH 3, 4, 5, and 5.5 were 0.10 × 10^−^^3^, 2.70 × 10^−^^3^, 5.40 × 10^−^^3^, and 3.6 × 10^−^^3^ Gy^−^^1^, respectively. The results showed that radiation sensitivity was enhanced by the influence of pH up to pH 5, and then decreased. The highest sensitivity of the PVA/AgNPs solution was found at pH 5, approximately 52.97, 1.97, and 1.49 times higher than pH 3, 4, and 5.5, respectively. Thus, the PVA/AgNPs solution with pH 5 was selected to develop our radiation sensor due to its linear dose response and high radiation sensitivity.

#### 3.2.2. Effect of AgNO_3_ Concentration

**Figure 4:**
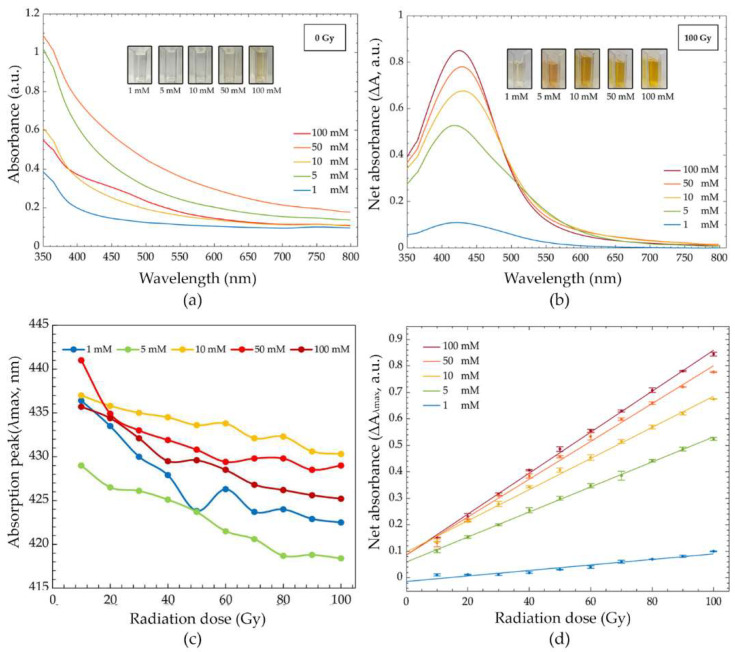
(**a**) Absorption spectra of the PVA/AgNPs solution before irradiation (1, 5, 10, 50, and 100 mM), (**b**) net absorption spectra after 100 Gy irradiation (1, 5, 10, 50 and 100 mM), (**c**) absorption peak (λ_max_) in the dose range of 0–100 Gy (1, 5, 10, 50, and 100 mM), and (**d**) dose response curves in the dose range of 0–100 Gy (1, 5, 10, 50 and 100 mM).

The effect of the AgNO_3_ concentration was investigated by varying concentrations (1, 5, 10, 50, and 100 mM) at pH 5. Before irradiation, the color of the solutions appeared colorless for 1, 5, and 10 mM, light yellow for 50 mM, and dark yellow for 100 mM (Figure 4a). These changes in color were consistent with the formation of AgNPs in the system. Increases in absorption intensity were observed as AgNO_3_ concentration increased both pre- and post-irradiation (Figure 4a,b). After exposure to gamma irradiation (Figure 4b), the color of the PVA/AgNPs solutions changed progressively with the radiation dose. These color changes agreed with increases in the absorption intensity of λ_max_ that indicated a greater presence of AgNPs. The slight red shift (Figure 4b) of the main peak reflected the increase in the diameter of nanoparticles when the AgNO_3_ concentration was increased. The large nanoparticle sizes with a high content of Ag^+^ depended on an increased ion association rate, high nanoparticles aggregation rate, and loss of polymer capping ability on the surface of nanoparticles [40]. Similar results (increase in nanoparticle size at a high Ag^+^ concentration) were observed by other researchers [15,29]. The blue shift phenomena with radiation was observed for all concentrations (Figure 4c). In addition, the reduction of FWHM at a high concentration was observed. This phenomenon is likely explained by the increased homogeneity of AgNPs’ size in the system. On the other hand, the large FWHM at a low AgNO_3_ concentration reflected the wide range of AgNPs’ size in the solutions. 

Figure 4d represents the dose response curve of different AgNO_3_ concentrations. The peak of each solution was at 422, 418, 430, 429, and 425 nm for 1, 5, 10, 50, and 100 mM, respectively. The estimated radiation dose based on the change of absorption intensity completely fit with the linear regression models for all concentrations. Pearson’s correlation coefficients were ~0.971 for 1 mM and >0.997 for 5, 10, 50, and 100 mM. The dose response curves show a marked dependence on the AgNO_3_ concentration and radiation dose. The radiation sensitivities of the PVA/AgNPs solution were 1.00 × 10^−^^3^, 4.70 × 10^−^^3^, 5.90 × 10^−^^3^, 7.10 × 10^−^^3^, and 7.70 × 10^−^^3^ Gy^−^^1^ for 1, 5, 10, 50, and 100 mM AgNO_3_, respectively. The differences in radiation sensitivity depended on the quantity of synthesized AgNPs in a solution. Sensitivity was enhanced approximately 4.55, 5.68, 6.90, and 7.47 times for 5, 10, 50, and 100 mM, respectively, when compared to 1 mM. The highest radiation sensitivity was found with 100 mM AgNO_3_. However, the abundance of Ag^+^ in the 100 mM AgNO_3_ solution turned the solution yellow before irradiation. The presence of background color prior to irradiation led to a poor ability to estimate radiation dose with the naked eye. It is important to note that we were able to observe and estimate the radiation dose with the naked eye up to 60 Gy for 50 mM and 40 Gy for 100 mM. Thus, 50 mM AgNO_3_ at pH 5 was selected for further development as a colorimetric radiation dosimeter due to its ability to be used in a qualitative dose assessment, linear dose response, and high radiation sensitivity.

### 3.3. Accuracy of PVA/AgNPs Radiation Sensor

**Figure 5:**
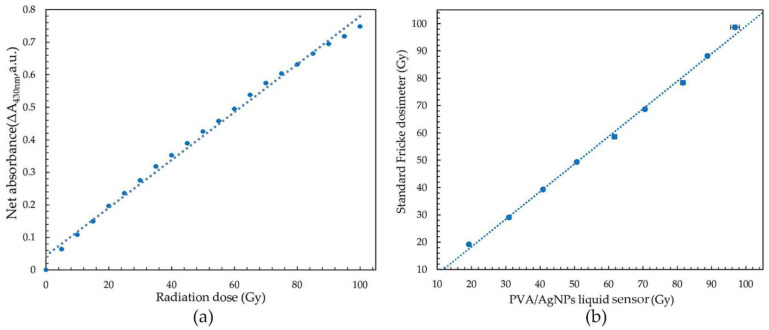
(**a**) Calibration curve of the optimized PVA/AgNPs sensor, and (**b**) the intra-class correlation coefficient (ICC) of the PVA/AgNPs sensor and standard Fricke dosimeter.

Calibration curves of the PVA/AgNPs sensor were established using 50 mM AgNO_3_ in 5% (*w/w*) PVA solution at pH 5. The sensor was subjected to 0–100 Gy (5 Gy intervals) of gamma rays (Figure 5a). The wavelength of 430 nm was selected due to the fact that it had the most change of absorbance after irradiation (to 100 Gy). There was a positive correlation between the change of the characteristic absorption peak and the received dose (*r* = 0.998, *n* = 20, *p* < 0.01). The radiation sensitivity of the PVA/AgNPs sensor was 7.2 × 10^−^^3^ Gy^−^^1^, while that of the standard Fricke dosimeter (High-Dosimetry Calibration Laboratory (HDCL), Office of Atoms for Peace, Bangkok, Thailand) was 3.5 × 10^−^^3^ Gy^−^^1^. 

The accuracy of dose measurements was estimated by the comparison with those from a standard Fricke dosimeter. Estimates were done in the range of 20 to 100 Gy, in accordance with the recommendation of the reference dosimeter (ASTME 51026:2015E). The ICC score (0.998) showed that there was excellent correlation between the PVA/AgNPs and the Fricke dosimeter (Figure 5b). In addition, the radiation sensitivity of the PVA/AgNPs sensor increased 2.06 times compared to the standard Fricke dosimeter in this range. The limit of detection (*LOD*) was calculated using Equation (1) to determine the lowest radiation dose detected by the PVA/AgNPs sensor [53],

(1) $$LOD=\frac{3.3\times SD}{m}$$

where “*SD*” is the standard deviation of absorption intensity and “*m*” is the slope of the calibration curve. It was found that the *LOD* of the PVA/AgNPs sensor was 13.40 Gy, which was lower than the *LOD* of the standard Fricke dosimeter.

### 3.4. Stability of PVA/AgNPs Radiation Sensor

**Figure 6:**
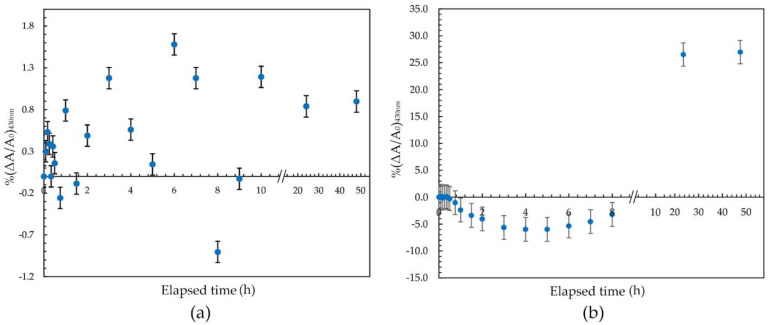
(**a**) Pre-irradiation (0 Gy) and (**b**) post-irradiation (100 Gy) stability of the PVA/AgNPs sensor during the first 48 h.

The stability of the radiation sensor was investigated by measuring the change in the absorption intensity at 430 nm before (pre-irradiation) and after irradiation, with 100 Gy (post-irradiation) during the first 168 h. The percentage change of the absorption intensity (at the 430 nm) was calculated by %(ΔA/A_0_)_430nm_, where ΔA was the net absorption intensity change and A_0_ was the absorption intensity of its first reading. The prepared solutions were kept in a dark environment at 25 °C and 50% relative humidity before measurement. Figure 6a shows the relationship between the percentage change of the absorption intensity and the storage time of the sensor before irradiation. The percentage change of the absorption intensity was less than 1.57% during the first 48 h. Then, responses increased significantly with the elapsed time. The measurement after 168 h was found to be 17.15%. The solution’s increase in absorption intensity at 430 nm without irradiation was most likely due to the continuous reduction of Ag^+^ to Ag^0^. Thus, in order to avoid errors of dose measurement from the early formation of AgNPs in the solution, the PVA/AgNPs sensor was used within 48 h of preparation. 

Post-irradiation stability of the PVA/AgNPs sensor was investigated. The solution showed high stability for up to 60 min, with the percentage change of intensity less than 2.38% (Figure 6b). The percentage absorption change increased ~0.4% per hour during the first 8 h, then increased sharply to approximately 1.20% per hour post-irradiation during the subsequent 168 h. The decrease in absorption intensity during the first 1~8 h might be due to the high mobility of polymer chains, resulting in the movement of AgNPs that allows crystals to aggregate and/or agglomerate and so reduce absorption intensity [50]. After more than 10 h post-irradiation, the absorption intensity sharply increased, indicating the process of back-oxidation of Ag^+^, Ag^0^, and Ag nanocluster [28] in the system. This back-oxidation would increase the number of AgNPs in the sensor by further agglomeration and/or an ion association process, resulting in increased absorption intensity during this period. This increase in absorption intensity related to storage time was similar to the trends reported by Soliman et al. [24] and Tadros et al. [23]. The increased signal at 430 nm confirmed that the effect of storage time was based on the amount of synthesized AgNPs. Thus, it was recommended that the sensor should be measured within the first 60 min after irradiation, minimizing the growth of AgNPs in the solution.

### 3.5. Uncertainty of Dose Measurement by the PVA/AgNPs Sensor

To confirm that the optimized PVA/AgNPs solution could reasonably be utilized as a gamma radiation sensor, the uncertainty of dose measurements was evaluated (Table 1). This term was defined as a parameter associated with the dispersion of the measured values. Typically, uncertainty can be grouped in to two categories known as “type A or random error (U_A_)” and “type B or systematic error (U_B_)”. Type A uncertainty is caused by random error, which increases the variation of replicate measurements. This type of uncertainty can be evaluated by the statistical analysis of repeated measurements. The effect of random error can be minimized by increasing the number of replications. On the other hand, type B uncertainty remains constant or its variation is predictable; for example, the effect of irradiation temperature on sensor response [54]. The uncertainty parameters associated with dose measurement of the PVA/AgNPs radiation sensor are discussed as follows.

Batch non-homogeneity (*U_batch_*) was assessed by irradiating six sets of PVA/AgNPs solution at different absorbed doses and analyzing within 60 min. Estimated uncertainty was calculated using the Equation (2) [54],

(2) $$U_{batch}=\sqrt{\frac{\sum_{i}\left(n_{i}-1\right){\left(\sigma_{i}\right)}^{2}}{\sum_{i}\left(n_{i}-1\right)}}$$

where *n_i_* is the number of replication and *σ_i_* is the standard deviation of the dose measurements at a given dose. The highest estimated uncertainty at 100 Gy was 0.9639% for the optimized PVA/AgNPs radiation sensor. 

The uncertainty of the calibration function (*U_cal_*) was used to fit the calibration curve, which directly affected the dose assessment. The uncertainty was calculated by using the Equation (3) [54],

(3) $$U_{cal}=\sqrt{\frac{\sum {\left(R\right)}^{2}}{n_{d}-n_{c}}}$$

where R is the residual of the calibration curve for each dose, n_d_ is the number of dosimeter readings, and n_c_ is the number of coefficients in the selected mathematical fitting function. 

Uncertainty due to the calibration function in this study was found to have the lowest value at the center of the selected dose range, which was 50 Gy (*U_cal_* = 0.1050%), and often increased at low doses due to the signal-to-noise ratio. Therefore, in order to cover uncertainties in the range of 13.4–100 Gy, the highest value of *U_cal_* at 10 Gy (=0.9177%) was used.

Pre- and post-irradiation stabilities of the PVA/AgNPs solution were additional sources of uncertainty and directly affected dose assessment. The pre-irradiation stability of the PVA/AgNPs solution in the controlled environment was less than 1.57% during the first 48 h. Moreover, the post-irradiation uncertainty was less than 2.38% within 1 h of irradiation. The standard uncertainties pre- and post-irradiation were 0.906% and 1.372% (based on dividing the stability with $\sqrt{3}$ divisor rectangular distribution), respectively. Thus, the expanded uncertainty of the PVA/AgNPs radiation sensor in the dose range of 10–100 Gy was 4.962% at a 95% confidence level. However, uncertainty improvement could be obtained by increasing the number of replicates in the calibration process. Results were in accordance with the recommendation that the uncertainty of routine dosimeters be less than 6% [55]. Thus, the PVA/AgNPs radiation sensor was successfully developed and has the potential to be a routine dosimeter due to its high sensitivity and accuracy in the dose range of 13.4–100 Gy.

## 4. Conclusions

A liquid radiation sensor based on poly(vinyl alcohol) doped with silver nitrate was investigated under different conditions in the range of 0–100 Gy of gamma radiation. The condition of 50 mM AgNO_3_ in 5% (*w/w*) PVA solution at pH 5 was selected as optimal due to its linearity, qualitative dose assessment, and high radiation sensitivity. The dose response curve showed excellent positive correlation (*r* = 0.998) between the increase in absorption intensity and the radiation dose. The accuracy of the PVA/AgNPs sensor was compared to the standard Fricke dosimeter in the range of 20–100 Gy. The ICC score showed excellent reliability between the PVA/AgNPs sensor and the Fricke dosimeter. In contrast, the PVA/AgNPs liquid radiation sensor had 2.06 times greater sensitivity compared to the standard reference dosimeter. The limit of detection of the liquid sensor was found to be 13.4 Gy. Moreover, overall uncertainty was found to be 4.962%, which is within the acceptable range of routine dosimeters (6%). Based on these results, we proposed that the PVA/AgNPs liquid radiation sensor was successfully developed and offers an alternative dosimeter for gamma radiation with high accuracy and sensitivity.

## Figures and Tables

**Table 1:** Sources of uncertainty associated with absorbed dose measurements by the developed PVA/AgNPs liquid dosimeter in the dose range of 10–100 Gy.

Uncertainty	Type of Uncertainty	Standard Uncertainty, %
Irradiation facility *^,^ **	B	1.316
Uncertainty of instrument ***	B	0.006
Sensitivity variation of instrument ****	A	0.006
$\mathrm{Batch}\;\mathrm{non}\text{-}\mathrm{conformity}\;(U_{batch}$)	A	0.936
$\mathrm{Uncertainty}\;\mathrm{of}\;\mathrm{calibration}\;\mathrm{function}\;\mathrm{fitting}\;(U_{cal})$	A	0.917
Post-irradiation stability	A	1.372
Pre-irradiation stability	A	0.906
Combined standard uncertainty (*σ*)		2.481
Expanded uncertainty (2*σ*)		4.962

* As quoted from calibration provided by High-Dosimetry Calibration Laboratory, Office of Atoms for Peace, Bangkok, Thailand. ** It includes decay correction, timer setting, temperature correction, repeatability, balance, and positioning effect. *** As quoted from the calibration certificate (Certificate NO: S2020/231) performed by Bangkok High Lab Co., Ltd., Bangkok, Thailand. **** It was estimated from the absorption measurements of an irradiated PVA/AgNPs sensor at 430 nm 100 times, while the standard cuvette was fixed at the sample position [1].

## Data Availability

Not applicable.
